# Epidemiological analysis of 556 procedures of open thoracoabdominal aortic aneurysm repair in the Public Health System in the largest Brazilian city

**DOI:** 10.31744/einstein_journal/2022AO6724

**Published:** 2022-03-17

**Authors:** Alexandre Maierá Anacleto, Marcia Maria Morales, Marcelo Passos Teivelis, Marcelo Fiorelli Alexandrino da Silva, Maria Fernanda Cassino Portugal, Nickolas Stabellini, Claudia Szlejf, Edson Amaro, Nelson Wolosker

**Affiliations:** 1 Hospital Israelita Albert Einstein São Paulo SP Brazil Hospital Israelita Albert Einstein, São Paulo, SP, Brazil.; 2 Faculdade Israelita de Ciências da Saúde Albert Einstein Hospital Israelita Albert Einstein São Paulo SP Brazil Faculdade Israelita de Ciências da Saúde Albert Einstein, Hospital Israelita Albert Einstein, São Paulo, SP, Brazil.

**Keywords:** Aortic aneurysm, Aorta, thoracic, Aorta, abdominal, Aneurysm, dissecting

## Abstract

**Objective:**

Despite the development of endovascular procedures, open repair remains the gold standard for the treatment of aortic thoracoabdominal aneurysms and some type B dissections, with well-established good outcomes and long-term durability at high-volume centers. The present study described and analyzed public data from patients treated in the public system in a 12-year interval, in a city where more than 5 million inhabitants depend on the Public Health System.

**Methods:**

Public data from procedures performed between 2008 and 2019 were extracted using web scraping techniques. The variables available in the database include sex, age, elective or emergency hospital admission, number of surgeries, in-hospital mortality, length of stay, and information on reimbursement values.

**Results:**

A total of 556 procedures were analyzed. Of these, 60.79% patients were men, and 41.18% were 65 years of age or older. Approximately 60% had a residential address registered in the municipality. Of all surgeries, 65.83% were elective cases. There were 178 in-hospital deaths (mortality of 32%). In the elective context, there were 98 deaths 26.78% *versus* 80 deaths (42.10%) in the emergency context (p=0.174). Mortality was lower in the hospitals that performed more surgeries. A total of USD 3,038,753.92 was paid, an average of USD 5,406.95 for elective surgery and USD 5,074.76 for emergency surgery (p=0.536).

**Conclusion:**

Mortality was no different between groups, and hospitals with higher volume presented more favorable outcomes. Specialized referral centers should be considered by health policy makers.

## INTRODUCTION

The definition of aneurysm is a localized and permanent dilatation of an artery at least 50% of its diameter. Thoracoabdominal aortic aneurysms (TAAA) are dilatations of the aortic segment that extends from the chest to the abdomen. They account for 10% of all aortic aneurysms.^(1-4)^ An observed incidence of approximately 5.9 cases per 100,000 persons per year over the recent decades led to efforts to improve the quality and the results of TAAA management.^(5-7)^ In Brazil, an incidence of 0.06% of TAAAs was reported in a screening study analyzing tomography exams of patients over 50 years of age.^(5)^ International guidelines currently recommend surgical intervention preventive of rupture for absolute aortic diameters of 5.5cm to 6.0cm.^(6)^ Unexpected rupture of TAAA is almost invariably fatal. However, even elective repair is associated with substantial risk, including death and life-altering complications such as paraplegia, permanent stroke, and dialysis-requiring renal failure.^(6)^

Thoracoabdominal aortic aneurysms therapeutic options include conventional open treatment, in use for over 50 years, and the more recent addition of endovascular therapy applicable for specific cases.^(4)^ Both techniques have advantages and disadvantages, with, however, the monetary burden inherent to the endovascular options rendering them prohibitive to the Brazilian Public Health System (SUS - *Sistema Único de Saúde*) and not regularly covered in the city of São Paulo, state of São Paulo, Brazil.

The early mortality rates for open TAAA repair vary with the extent of the replaced aorta as well as other patient-specific variables. The mortality rates at experienced centers range from 2% to 21%, depending on the anatomic classification of the aneurysm,^(7)^ with global rates as low as 5 to 12% in selected centers.^(8)^ In hospitals with lower procedure volumes, nonetheless, rates of survival and good functional outcomes were significantly lower than expected, with mortality rates reaching over 20%.^(9)^

São Paulo is the largest city in Brazil, with a population over 12 million people. Seven million of these have private health care. However, as many as 5 million are totally dependent on the public system.^(10-13)^ Almost all procedures performed to treat TAAA in the public system are open surgeries, and a small fraction for aortic aneurysms secondary to dissection are open surgery as well.

Large series of TAAA have been analyzed in the USA and Europe.^(14-16)^ Mortality from aortic aneurysms and dissection in São Paulo has been previously analyzed through the evaluation of death certificates over a 24-year interval, and this study found that aneurysms are the 6^th^ cause of death in the city;^(14)^ to the best of our knowledge, however, there are no studies focusing on TAAA surgical repair outcomes in Brazil spanning over 10 years.

## OBJECTIVE

The objectives of this study were to evaluate the frequency of thoracoabdominal aortic aneurysms surgical procedures performed by the Brazilian Public Health System in São Paulo between 2008 and 2019, assessing in-hospital mortality, length of stay, and reimbursement values paid according to the type of surgical procedure performed (elective or emergency).

## METHODS

All patient data was collected from a platform belonging to the Municipal Health Department of São Paulo called *Departamento de Informática do Sistema Único de Saúde do Brasil* (DATASUS). This platform lists only hospitals accredited as high complexity referenced centers for the SUS. Only open TAAA correction procedures performed by the SUS between 2008 and 2019 were selected to be evaluated. Procedures were selected in accordance with the SUS System of Procedures and Medicines Coding System, using the following codes to identify patients subjected to open aortic repair for TAAA in both elective or emergency contexts: TAAA/dissection repair (code: 04.06.01.013-7) and thoracoabdominal aortic aneurysmectomy (code: 04.06.02.005-1). To avoid including congenital malformations of the great arteries, all procedures classified as Q25 by the International Classification of Diseases were dismissed.

The DATASUS database permits evaluation of variables such as patient demographics, reimbursement values, procedure type (elective or emergency), in-hospital mortality, intensive care unit (ICU) length of stay, and hospital length of stay.

To facilitate and expedite data collection, automatic navigation codes were used with programming assistance. These codes were programmed in the Python language (v. 2.7.13, Beaverton, Oregon, USA) using the Windows 10 Single Language operating system. Data collection, platform field selection, and table adjustment were performed using the Selenium WebDriver packages (v. 3.1.8, Selenium HQ, various contributors around the world) and pandas (v. 2.7.13, Lambda Foundry, Inc. and PyData Development Team, New York, USA). The web scraping code has a main structure with 14 search phases (Appendix A) adaptable to the different filters available on the platform. Mozilla Firefox browser (v. 59.0.2, Mountain - California - USA) and WebDriver.GeckoDriver (v 0.18.0, Mozilla Corporation, Bournemouth, England) were used.

The navigation codes enabled a simpler and quicker use of the available public data, thus permitting the analysis of a larger number of procedures and years.

After collection and cleaning, all data was organized in a spreadsheet using the Microsoft Office Excel 2016^®^ software (v. 16.0.4456.1003, Redmond - Washington - USA). The main table was formatted to display the following subdivisions: number of procedures performed in each of the establishments (ranked in descending order), total number of operated patients, in-hospital mortality (absolute and percentage), length of stay, and reimbursement values paid. The amounts paid in *Reais -* Brazilian official currency (BRL) were converted into American dollars (USD) using the rating of December 31, 2012 (which is the intermediate date between the first and last data analyzed),^(15)^ in which USD 1.00 was equivalent to BRL 2.0429. The analyzed hospitals were numbered, in descending order, by the total number of procedures performed.

The frequency of procedures was analyzed by procedure type, by year; the mortality was assessed by procedure type, by hospital unit; the length of hospital stay, by procedure type; and procedure cost, by procedure type, by hospital unit.

For the statistical analysis, the following tests were used: the χ^2^ test was applied as a trend test, to investigate whether there was a tendency for change over the years in the number of procedures for each procedure type (elective and emergency); the Mann-Whitney test was used to compare mortality, time of hospitalization, and reimbursement values paid between the elective and emergency groups. For all tests, the level of statistical significance was p=0.05.

This study was approved by the Institutional Review Board (Protocol # 3067-17) and the Ethics Committee of *Hospital Israelita Albert Einstein* (CAAE: 35826320.2.0000.0071, opinion # 4.321.508) and was deemed exempt from the informed consent requirement because the data was non-identifiable.

## RESULTS

A total of 556 procedures were performed in the city of São Paulo from 2008 to 2019. Most of the patients treated were men (n=338, 60.79%) and 39.20% were women (n=218). A total of 222 procedures (41.18%) were performed in elderly patients, aged 65 years and over; whereas 317 were younger (58.81%). Age was not documented in 17 cases. Almost two-thirds (n=325, 60.07%) of the operated subjects had a registered residential address in the city of São Paulo, whereas 39.93% (n=216) emigrated from other cities and provenance was unknown in 15 cases.

Table 1 shows the number of procedures per year, according to type (elective or emergency). Elective procedures were more common (65.83%), and the trend test demonstrated no tendency toward a shift in this distribution throughout the years (p=0.230). An overall reduction on the total number of procedures along the evaluated years was observed.

**Table 1:** Absolute and relative frequency of procedures for thoracoabdominal correction according to degree of emergency from 2008 to 2019

	Elective n (%)	Emergency n (%)	Total	p value*
2008	37 (52.86)	33 (47.14)	70	0.230
2009	50 (58.82)	35 (41.17)	85	
2010	53 (64.63)	29 (35.36)	82	
2011	41 (71.93)	16 (28.07)	57	
2012	42 (72.41)	16 (27.59)	58	
2013	29 (82.86)	6 (17.14)	35	
2014	24 (85.71)	4 (14.28)	28	
2015	29 (67.44)	14 (32.56)	43	
2016	14 (70)	6 (30)	20	
2017	22 (64.70)	12 (35.29)	34	
2018	20 (74.07)	7 (25.92)	27	
2019	5 (29.41)	12 (70.59)	17	
Total	366 (65.83)	190 (34.17)	556	

* p value from trend χ^2^ test.

Table 2 shows the mortality per hospital unit by procedure type. There were 178 in-hospital deaths (32%). Mortality during hospitalization for emergency procedures was higher than for elective procedures, (42.10% *versus* 26.78%), although this was not a statistically significant difference (p=0.174). The hospital with the highest number of surgeries (n=191) had the lowest in-hospital mortality (12.04%).

**Table 2:** Absolute and relative frequency of procedures (%) and mortality (%) divided by hospital unit

			Open procedures			

Hospital	Total procedures	Total mortality n (%)	Elective procedures	Mortality n (%)	Emergency procedures	Mortality n (%)
1	191	23 (12.04)	189	23 (12.17)	2	0
2	98	46 (47.95)	98	46 (46.94)		
3	79	18 (22.78)	7	3 (42.85)	72	15 (20.83)
4	64	29 (45.31)	10	0	54	29 (53.70)
5	54	24 (44.44)	22	8 (36.36)	32	16 (50)
6	35	19 (54.28)	26	11 (42.31)	9	8 (88.89)
7	30	17 (56.66)	11	6 (54.54)	19	11 (57.89)
8	4	2 (50.0)	2	1 (50)	2	1 (50)
9	1	0	1	0		
Total	556	178 (32.01)	366	98 (26.77)	190 (34.17)	80 (42.10)
p value*				0.174		

* p value from Mann-Whitney test.

Table 3 shows the length of hospital stay. Most procedures were associated with a hospital stay longer than 15 days (n=256, 46.06%). However, attributing the length of stay of less than two days to early mortality, can infer that, among the survivors, approximately 55% remained hospitalized for more than 15 days and almost 20% for more than 30 days.

**Table 3:** Absolute and relative frequency of procedures according to the number of days (time) of hospitalization

	Length of stay					

	<2 days	3-7 days	8-14 days	15-30 days	>30 days	p value*
Elective	39 (10.65)	50 (13.66)	84 (22.95)	120 (32.79)	73 (19.94)	<0.001
Emergency	58 (30.52)	24 (12.63)	45 (23.68)	48 (25.26)	15 (7.91)	
Total	97 (17.45)	74 (13.30)	129 (21.20)	168 (29.68)	88 (15.55)	

* p value from Mann-Whitney test.

Table 4 shows the values in dollars paid by the SUS by hospital unit.

**Table 4:** Values paid by the Brazilian Public Health System in American dollars per hospital unit

Hospital	Total amount	Total amount paid per procedure type		Average paid per procedure	

		Elective	Emergency	Elective	Emergency
1	1.305.058,54	1.291.834,29	13.224,25	6.835,10	6.612,12
2	575.991,53	575.991,53		5.877,46	
3	413.001,45	49.236,21	363.765,24	7.033,74	5.052,30
4	323.591,34	51.345,63	272.245,71	5.134,56	5.041,59
5	220.176,72	94.614,24	125.562,48	4.300,65	3.923,83
6	125.021,54	94.261,38	30.760,16	3.625,44	3.417,80
7	46.648,46	104.754,22	151.402,68	4.240,77	5.513,38
8	23.374,34	11.449,70	11.924,64	5.724,85	5.962,32
9	5.890,01	5.890,01		5.890,01	
Total	3.038.753,92	2.279.377,21	968.885,16	5.406,95	5.074,76

Cost per patient of urgent procedure was not statistically different than elective surgery (p value=0.536), Mann-Whitney test.

A total reimbursement of USD 3,038,753.92 was paid for all hospitals for open TAAA correction procedures during the 12 years. Elective procedures were paid, in average, USD 5,406.95 and emergency procedures, in average, USD 5,074.76, which incurred no statistical significance (p=0.536).

## DISCUSSION

The SUS is a universal, equitable, and comprehensive tax-funded government system which covers health care for the entire country’s population. Seventy-five percent of the population are exclusively covered by the SUS.^(16)^ The remaining portion (25%) makes use of the private supplementary health system, in which costs are covered individually either by the user or their employer.^(17)^ In the city of São Paulo, however, this pattern is not maintained, since approximately 58.33% of the population (7 million people) have access to private health care, while the remaining 5 million people depend exclusively on the Public Health System. As a rule, highly costly and complex endovascular procedures (such as fenestrated or branched endoprosthesis) are not covered by the SUS; anecdotal cases have been performed by donation of materials, but constitute an absolute minority.

The analyzed sample was comprised mostly of men patients (n=338, 60.79%), and 39.92% of all patients were over 65 years old. This demographics are in accordance with the expected findings for TAAA populations as seen in previous studies.^(9,11)^

Considering an incidence of 5.9 cases per 100,000 habitants per year,^(1)^ an estimate can be made that approximately 295 new cases of TAAA would be diagnosed by the public system in the city of São Paulo each year. The yearly average of operated patients in the nine evaluated institutions in the past 12 years was 46, which represents approximately one-seventh of the estimated diagnosed cases (without considering aortic dissections). Although the correct proportion of diagnosed cases with definite surgical indication (>6cm) is unknown, it may be assumed that the intervention rate is lower than expected. Moreover, as almost 40% of patients operated in this city come from other cities, it is possible that most cases of thoracoabdominal aortic diseases are not operated on time, thus, a large proportion of patients will likely present rupture and death.

Furthermore, a tendency of reduction in the annual number of procedures was observed throughout the studied years, which may be attributed to several factors, such as the widespread of endovascular techniques, the reduction of training in complex conventional surgery for vascular surgery residents,^(18)^ and late disease discovery, leading to an overall worse clinical status of patients and higher surgical risk and availability of endovascular techniques in some public institutions which may opt for these technique instead of the conventional approach.

Of all surgeries, 65.83% were performed in an elective scenario and 34.17% were emergency cases. The proportion of emergency cases reported in other large studies is not negligible - Coselli et al. reported 21.8%,^(19)^ and Gopaldas et al. reported 15.9% for the endovascular group.^(20)^ This difference may be a result of coding errors, but may also arise from a perspective of relative prohibitive mortality for elective procedures, with some surgical teams often choosing to retain a conservative approach for most aneurysms, operating only on emergency cases.

Data from several retrospective surveys indicate that for patients in whom surgery is delayed for thoracic aneurysms, rupture was the most common cause of death, with death rates ranging from 42% to 74%.^(21)^

For this reason, it is reasonable that, in a well-structured system, surgical treatment should be instituted immediately after diagnosis and elective procedures would, therefore, surpass emergency procedures in volume.

It would be interesting to correlate these findings with necropsy results (of both intact aneurysms, in patients who died from other causes, and patients who died from ruptured aneurysms), as proposed by Lobato et al.,^(22)^ for a more comprehensive notion on the impact of this elective *versus* emergency proportion.

In this sample, the mortality rates were evaluated by hospital unit to infer a possible correlation of outcomes and surgical volume.

In referral centers for the treatment of TAAA in the United States, the emergency admissions have a significant influence in mortality rates, which double when compared to that of elective surgeries, even in the best referral centers worldwide, with emergency mortality ranging from 12.2% to 20%, *versus* 6.2% to 6.7% for elective cases,^(8,19)^ which should be the gold standard for the management of this disease. Some authors attribute this difference to the hemodynamic instability secondary to rupture as well as the poor conditions of preoperative preparation of the emergency patients. It is also possible that patients with imminent rupture belong to a high-risk subgroup, having been denied elective repair due to associated comorbidities.^(8)^ The mortality rates in this sample were twice as high for emergency repairs, and a lot higher than the rates reported by North-American referral centers (42.10% *versus* 26.78%).^(8,19)^ Interestingly, no significant difference was found between groups (p=0.174), which may be explained because 44.4% of the evaluated hospitals in this sample performed two or less emergency procedures, which may have evened the statistical difference in mortality rates between groups.

Although a direct correlation cannot be made, it is assumed that the cases which demanded very short hospitalization periods (<2 days) can be considered as early in-hospital mortality. In the present sample, 97 patients (17.45%) were hospitalized for two or less days, which represents 54.49% of the overall mortality.

The hospital with the largest number of surgeries in this sample (n=191) had the lowest in-hospital mortality (12.04%). Some recent reports demonstrate that provider caseload volume is a valid predictor of postoperative mortality and complications after repair of intact TAAAs.^(23,24)^ Cowan et al. documented that patients with TAAAs treated at a high-volume hospital and by a high-volume surgeon (defined as a median of 12 cases per hospital per year and, seven cases per surgeon, per year) had a 42% and a 58% reduction in mortality, respectively.^(9)^ These findings further suggest that patients, at an acceptable surgical risk, with intact TAAAs may benefit from referral to experienced centers.^(9)^

In this study, most of the patients were hospitalized for more than 15 days (n=256; 46.04%). Patients with less than two days of hospital stay are considered as early in-hospital deaths (n=97 patients; 7.44%), as many as 55% of the surviving cases were hospitalized for more than 15 days, and almost 20% more than 30 days, reflecting the complexity of postoperative management.

According to Cowan et al., the hospital and surgeon procedure volume has a direct influence on the length of hospital stay.^(9)^ In their study, the authors found a significant difference in the early postoperative death rate (27.6% for high-volume hospitals, as opposed to 42.3% for low volume hospitals), and a significant difference in hospital length of stay among the surviving patients between high and low volume hospitals. The authors attribute these findings to differences in the perioperative management of these patients which may reflect a more complicated postoperative course depending on the expertise of the team.^(9)^

The cost analysis demonstrates a governmental investment of USD 5,406.95 per elective open procedure, and USD 5,074.76 per emergency open procedure. This parity in values between groups is due to the fact that initial costs are indeed similar (surgical materials, human and hospital resources allocation) and would likely vary in association with a longer ICU and hospital length of stay. However, emergency patients are likely to die more precociously, and this early mortality may have likened the costs between groups.

A comprehensive analysis of the profile of thoracic and TAAA corrections in the United Kingdom by Field et al. determined that a population of roughly 50 million people in England demands four to five specialized centers.^(25)^ Inferring these findings to the Brazilian population of about 200 million people, 16 qualified centers would be needed to adequately support open TAAA treatment in this country. An effective policy would be the establishment of a few national referral centers for the treatment of TAAA, following the strategy previously determined by the SUS for the heart and liver transplant services. Providing regional specialized centers for the treatment of TAAA would likely contribute to reduce the high mortality and morbidity rates and guide the rationalizing of disposable resources. The fact that almost 40% of patients operated in São Paulo are from other municipalities may be representative of some kind of referencing, but the fact that a few centers have very few surgeries may mean referencing is not yet optimized. Specialized centers should ideally encompass comprehensive multidisciplinary teams with expertise in open surgery, including the use of extracorporeal support and endovascular approaches, which could ensure that all appropriate treatment regimens are offered to all patients.

The critical focus on cost reduction in health care, particularly in Public Health Systems, shows it is time to carefully assess methods to reduce the cost of treating complex aortic aneurysms. The new emerging endovascular technologies face resistance from hospitals and insurance providers. Earlier studies on TAAA showed an increased cost of open repair related to the long length of stay and hospital-related charges, such as medications and laboratory testing. On the other hand, costs related to endograft are accountable for most of the spending in endovascular TAAA repair.^(26)^

### Limitations

The present study is primarily limited as for the data source. Data was obtained from an administrative database that is subject to coding errors, especially due to the constraints imposed by the SUS coding system. Dissections and aneurysms are not separated on the database, therefore they were analyzed as one disease. However, dissections subjected to operations are probable those that evolved with dilation and may be considered aneurysms. On the other hand, as surgical coding is different for open and endovascular treatment of aortic dissection (and further divided for aortic disease that involves the aortic arch), only the open surgical cohort for type B aortic dissection was analyzed in this manuscript.

Secondly, the database does not include data referring to readmissions, reinterventions, or long-term outcomes.

Certain information is displayed in the database in a summarized manner, such as length of hospital stay, distributed in groups, which does not permit a more accurate evaluation with proper median calculation.

Furthermore, variables related to the severity of rupture, such as whether or not patients were unstable at the time of presentation, and complex anatomy of specific cases are not registered in the database. This study was no able to better stratify the patients for risk-adjusted analysis due to the lack of such information.

The variable length of ICU stay was available within the platform, but because it is subject to several biases such as reduced length of stay because of early mortality, or contingency on availability of beds for discharge, this variable was not included in this analysis.

Finally, the cost analysis is restricted by the fact that the Public Health System compensation table is often subpar to the actual amount spent on the procedures, meaning that the hospital real expenditure per procedure may in fact have surpassed the values documented by the public database.

For these reasons, clinical decisions should not be based solely on the outcomes reported in an administrative database, but supported by clinical judgment and adequate standards of care.

Despite all limitations, this study shows a comprehensive analysis of the management of TAAA in the Public Health System in the largest urban center in Brazil, encompassing a 12-year interval. This study grants a representational assessment of a real-world sample of open surgery for TAAA patients, and presents a useful tool for better understanding of a public system and guidance for the allocation of health funds.

## CONCLUSION

The frequency of thoracoabdominal aortic aneurysms correction procedures performed in the city of São Paulo by the Brazilian Public Health System between 2008 and 2019 was subpar to the expected number of thoracoabdominal aortic aneurysms, suggesting that a large proportion of patients remain undiagnosed or untreated.

Elective procedures were almost twice as frequent as their emergency counterpart, in a worse proportion than that reported by reference centers. No difference was found as for mortality, length of hospital stay, or reimbursement values between the elective and emergency groups. Lower mortality was associated with higher volumes of surgery.

The mortality rate was higher in this sample compared to those reported in most large-scale studies. However, hospitals with the highest operation volumes had the lowest mortality rates.

This is large-scale study provides an initial examination of the descriptive profile of the open surgery management of thoracoabdominal aortic aneurysms in the city of São Paulo and may represent appropriate evidence to guide monetary resource allocation in the Public Health System.
